# Supplementary material for: Combinatorial Therapy: Targeting CD133+ Glioma Stem-like Cells with a Polysaccharide–Prodrug Complex Functionalised Gold Nanocages
Source: Biomedicines. 2024 Apr 23;12(5):934. doi: 10.3390/biomedicines12050934 (PMC11117750; doi:10.3390/biomedicines12050934)
Supplement: Supplementary file 1 [file biomedicines-12-00934-s001.zip › biomedicines-2891955-supplementary.pdf]

Supplementary Information

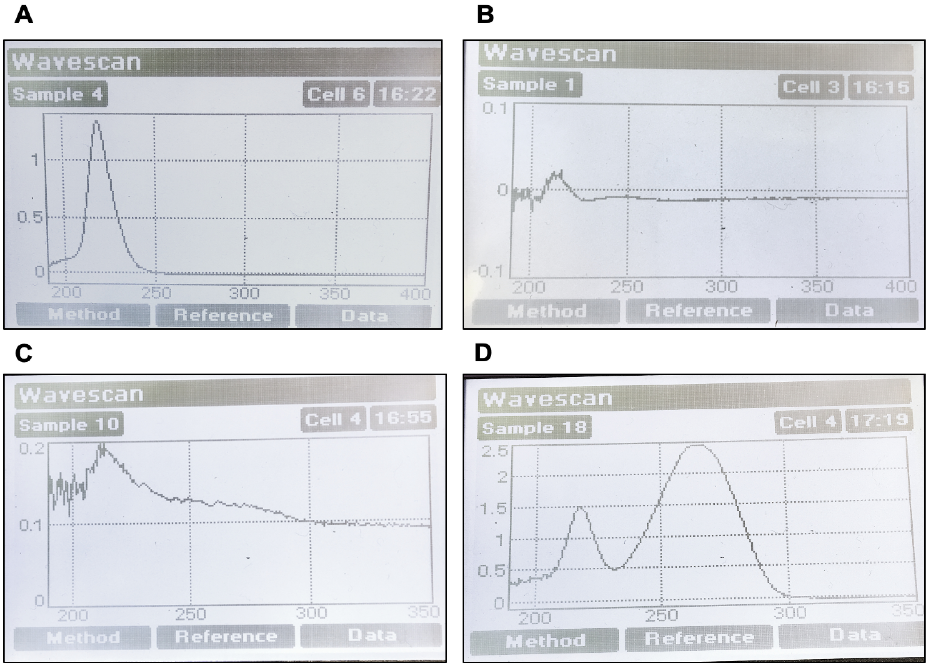

Figure S1: Qualitative analysis- UV-Vis spectroscopic measurement of 5FU release profile of from TMFG nanoparticles. **A,** 5FU spectra; **B,** PBS (pH 7.4)- day 13; **C,** PBS (pH 3.15)- day 13; **D,** ABS (pH 3.76)- day 13.

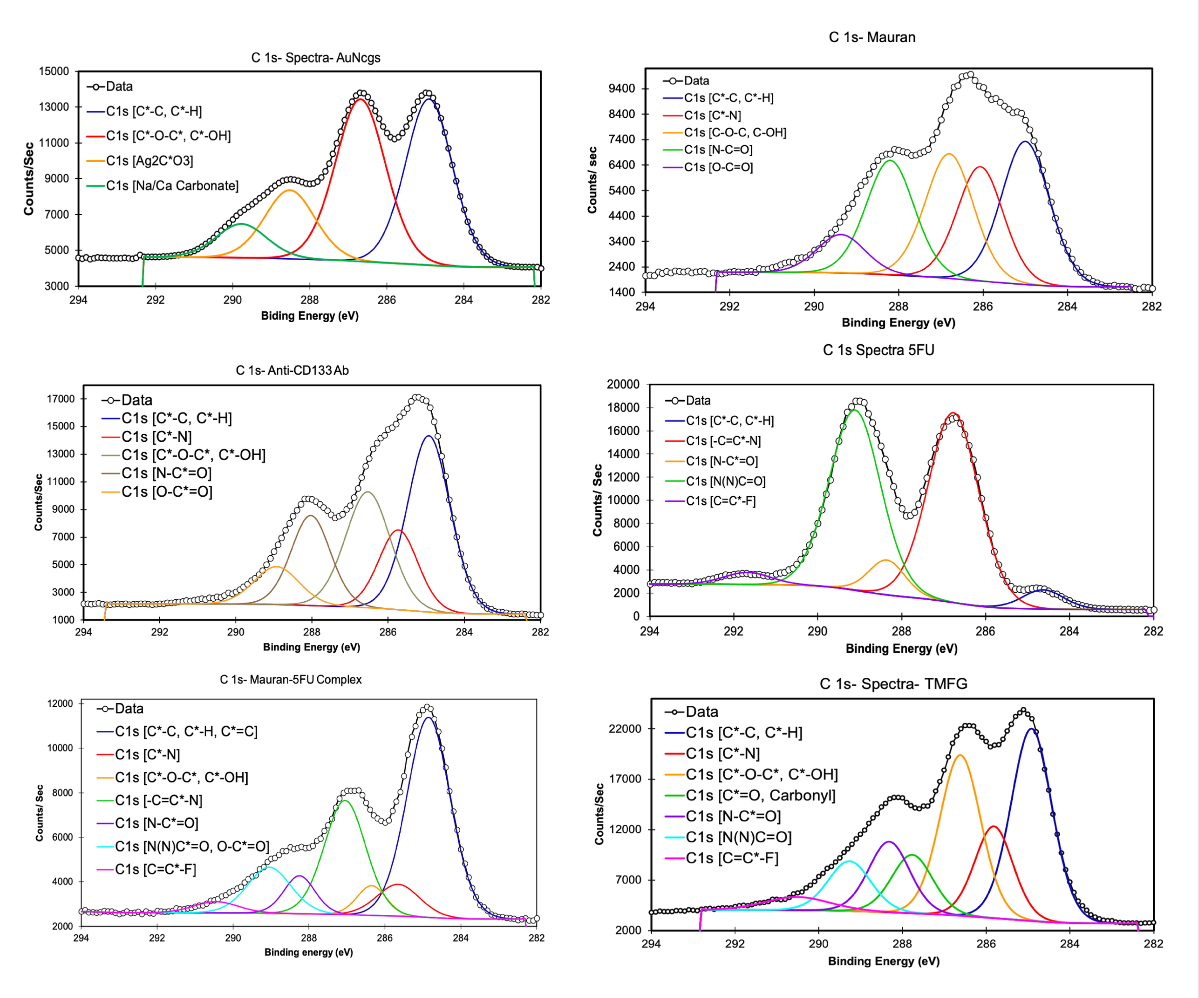

Figure S2: High resolution C 1s XPS spectra of **A,** AuNcgs; **B,** Mauran; **C,** Anti-CD-133 antibody; **D,** 5FU; **E,** Mauran-5FU complex; **F,** TMFG nanoparticles
